# On explicatures, cancellability and cancellation

**DOI:** 10.1186/s40064-016-2789-x

**Published:** 2016-07-19

**Authors:** Gregor Walczak

**Affiliations:** University of Münster, Germanistisches Institut, Schlossplatz 34, 48143 Münster, Germany

**Keywords:** Explicature, Cancellability, Cancellation, Pragmatic inferences

## Abstract

Within the Gricean framework only what is conversationally implicated is cancellable, whereas what is conventionally implicated and what is said cannot be cancelled without giving rise to contradiction. In the relevance-theoretic framework, however, the question is whether explicatures, which replace the Gricean notion of what is said, are cancellable. In recent years, various objections to the cancellability of explicatures have been raised. The aim of the present paper is to demonstrate that these objections are due to a misinterpretation of the Gricean cancellability test. In particular, they disregard the fact that this test is merely one of several diagnostic tools that are used by Grice to distinguish between conventional and conversational implicatures. Once we have recognized the essence of the cancellability test, the objections to the cancellability of explicatures turn out to be unwarranted.

## Background

Within his framework, Grice differentiates between certain kinds of implicatures such as conventional implicatures, generalized conversational implicatures and particularized conversational implicatures. However, keeping apart these different kinds of implicatures is not always that easy. In particular, the distinction between conventional and generalized conversational implicatures proves to be difficult in that generalized conversational implicatures “are relatively independent of context and therefore can rather easily be confused with conventional implicatures since they are constantly associated with particular linguistic forms” (Sadock 1978: 283). However, Grice maintains that there are certain tests which help to differentiate between conventional and conversational implicatures and which can be used for identifying conversational implicatures (see Huang 2014: 39–43). Probably the best known of these tests is the so-called cancellability test. As Blome-Tillmann points out, this test “has been brought to bear not only in linguistics and the philosophy of language but also in areas as diverse as ethics, epistemology, metaphysics and the philosophy of mind” (Blome-Tillmann 2008: 156).^1^ So let us take a look at what Grice tells us about the cancellability test:

You will remember that a putative conversational implicature that *p* is explicitly cancelable if, to the form of words the utterance of which putatively implicates that *p*, it is admissible to add *but not p*, or *I do not mean to imply that p*, and it is contextually cancelable if one can find situations in which the utterance of the form of words would simply not carry the implicature. (Grice 1978: 115–116)

As we can see, Grice draws a distinction between two types of cancellability which are called explicit cancellability and contextual cancellability. Explicit cancellability is given when one can cancel the implicature by amending a cancellation clause. However, the amendment of this clause must be “admissible” (ibid.: 115) and may not result in “logical absurdity” (Grice 1981: 186) or “linguistic offense” (ibid.). That is, the amendment of the cancellation clause may not give rise to the truth-theoretic relation of contradiction (see Cappelen 2000: 3). This is why cancellations of conventional implicatures are infelicitous, whereas cancellations of conversational implicatures are felicitous. Let us take a look at some examples of explicit cancellations:

1. She was poor but she was honest. #And poor people are usually honest.

The attempt to cancel the conventional implicature in (1) gives rise to contradiction, since we first make use of a certain form of words and then try to take it out again instead of omitting it in the first place (see Hagemann 2011: 215). In contrast, cancellations of conversational implicatures do not give rise to contradiction. For instance, imagine a situation in which someone is out of petrol. Even if the speaker in (2) implicates that the garage round the corner is open and has petrol to sell, this implicature can be cancelled by the amendment of a cancellation clause:

(2) There is a garage round the corner. But it’s closed.

So far, we have been concerned with cancellability in terms of explicit cancellability. Contextual cancellability, however, is a completely different matter. It is given when one can imagine an utterance context in which the utterance does not carry the implicature in question. Accordingly, contextual cancellations of conventional implicatures are infelicitous, whereas contextual cancellations of conversational implicatures are felicitous. To illustrate, consider once again the examples aforementioned. Given that the conventional implicature in (1) depends on the conventional meaning of the linguistic expression *but*, the implicature will be the same in any utterance context. This is why it cannot be cancelled by means of contextual cancellation. In contrast, the conversational implicature in (2) is contextually cancellable. For instance, we may imagine an utterance context in which the hearer just wants to know where she can find a garage. In this case, saying that there is a garage round the corner would not carry the implicature that the hearer can buy some petrol there. All the speaker conveys by her utterance is that there is a garage round the corner.

It is important to note that the two types of cancellability rely on two types of cancellation which are called explicit and contextual cancellation. In a sense, these two types of cancellation are rather different. While explicit cancellation pertains to a particular utterance context, contextual cancellation refers to a sentence uttered and the meanings that can be communicated with it in different utterance contexts. Accordingly, Jaszczolt maintains that explicit cancellation is an empirically verifiable phenomenon, whereas contextual cancellation is just a kind of thought experiment (see Jaszczolt 2009: 261). However, in what follows I argue that this view on the two types of cancellation is highly misleading and that both of them should be considered thought experiments: either we have to imagine a cancellation clause which cancels the implicature, or we have to imagine an utterance context in which the utterance does not give rise to the implicature.^2^ This view on explicit and contextual cancellation accounts for the fact that both are part of the Gricean cancellability test which is nothing else than one of several diagnostic tools used to distinguish between conventional and conversational implicatures.

However, there is dissent concerning the general conception of the cancellability test. In particular, it is sometimes argued that this test has to be understood as a conjunction of explicit and contextual cancellation which is why any conversational implicature has to be explicitly as well as contextually cancellable; otherwise we are not dealing with a case of conversational implicature (see Blome-Tillmann 2008: 157; Weiner 2006: 128). The problem with this view is that Grice merely mentions that the cancellation of an implicature may take two different routes: it may be cancelled explicitly or it may be cancelled contextually. Correspondingly, it is sufficient for a conversational implicature to be cancellable in at least one of the two ways aforementioned (see Jaszczolt 2009: 263).^3^

There is one last point that should be made with regard to the cancellability test. Given that this test meets certain problems, Grice does not “regard the fulfillment of a cancelability test as decisively establishing the presence of a conversational implicature” (Grice 1978: 116). Rather, the test indicates that we are possibly dealing with a conversational implicature (see Geurts 2010: 20). A first problem mentioned by Grice pertains to cases in which the speaker uses a form of words loosely:

One way in which the test may fail is connected with the possibility of using a word or form of words in a loose or relaxed way. Suppose that two people are considering the purchase of a tie which both of them know to be medium green; they look at it in different lights, and say such things as *It is a light green now*, or *It has a touch of blue in it in this light.* Strictly (perhaps) it would be correct for them to say *It looks light green now* or *It seems to have a touch of blue in it in this light*, but it would be unnecessary to put in such qualificatory words, since both know (and know that the other knows) that there is no question of real change of color. (Grice 1978: 116)

Since the tie does not really change its colour, part of what is meant is cancelled contextually in such cases (see Hirschberg 1991: 29). A second problem noticed by Grice refers to the use of such words as *see*:

If we all know that Macbeth hallucinated, we can quite safely say that Macbeth saw Banquo, even though Banquo was not there to be seen, and we should not conclude from this that an implication of the existence of the object said to be seen is not part of the conventional meaning of the word *see*, nor even (as some have done) that there is one sense of the word *see* which lacks this implication. (Grice 1978: 116)

The problems discussed by Grice prove that there are meanings which pass the cancellability test without being conversational implicatures.^4^ For this reason, the test provides only some evidence in favour of the presence of a conversational implicature which in turn forces us to use not only this test, but also other tests that are mentioned here and there in the pragmatics literature (see Levinson 1983: 119).

## The notion of explicature

Given that the present paper deals with the question of whether explicatures should be considered cancellable, we have to elucidate the notion of explicature. In this regard, it makes sense to shed some light on the Gricean framework which is based on the distinction between what is said and what is implicated. While the former is a matter of semantics (and merely affected by the pragmatic processes of disambiguation and reference assignment), the latter is a matter of pragmatics. However, this traditional distinction between semantics and pragmatics has been questioned, because what is said turned out to depend much more on pragmatic processes than originally assumed. Among these processes are not only reference assignment and disambiguation but also various enrichment and adjustment processes (see Clark 2013: 176). In the pragmatics literature, this insight is known under the name of the *semantic underdeterminacy thesis* which states that linguistic meaning (i.e. the semantic content of the sentence uttered) generally underdetermines what is said which is why the hearer has to resort to pragmatic inferencing in order to obtain the proposition directly communicated by an utterance (see Bach 2007: 30; Borg 2012: 75; Carston 2002: 19; Recanati 2004: 58; Soames 2010: 155). To illustrate, consider the following example:

(3) a. The princess is late.
  b. The princess is late for the party.

Suppose someone wants to communicate (3b) by uttering (3a). In this case the uttered sentence does not express a complete proposition which is why the hearer has to undertake a process of pragmatic inferencing in order to work out the proposition the speaker wants to communicate. Matters are different in the following case:

(4) a. Ramona has nothing to wear.
  b. Ramona has nothing appropriate to wear to a wedding.

Here, (4a) actually expresses a complete proposition but not the one the speaker wants to communicate. This is why the hearer has to pragmatically infer (4b). Both examples demonstrate the semantic underdeterminacy of what is said and thus the need of pragmatic processes in order to determine the proposition directly communicated by an utterance.

As one of several competing pragmatic theories, relevance theory acknowledges the semantic underdeterminacy of what is said but replaces this notion with the notion of explicature. An explicature is an explicitly communicated proposition which is a development of a logical form encoded by the utterance (see Sperber and Wilson 1986: 182). According to this definition, the main feature of an explicature is that it is recovered by both linguistic decoding and pragmatic inferencing. While the former pertains to the linguistic expressions used, the latter pertains to the pragmatic inferences that are involved in the development of the logical form encoded by the utterance (see Carston 2002: 117). Following this definition, we can say that utterances of (3a) and (4a) can be used to convey the explicatures in (3b) and (4b).

## Arguments in favour of cancellable explicatures

Now, let us turn to the question of whether explicatures should be considered cancellable. As we have seen, explicatures originate from two distinct sources, the linguistic expressions used and the utterance context, and they are derived depending on these sources, by linguistic decoding and by pragmatic inferencing (see ibid.). What does that mean for the question of cancellability? Much depends on how we understand pragmatic inferencing. In the pragmatics literature, it is common practice to distinguish between two types of inferencing. On the one hand, demonstrative inferencing is a process whereby the premises warrant the conclusion which is why the conclusion cannot be cancelled without giving rise to contradiction; on the other hand, non-demonstrative inferencing is a process whereby the premises do not warrant the conclusion, because they are merely probably true. To that effect, the conclusion can be cancelled without giving rise to contradiction. In view of this, pragmatic inferencing clearly falls in the category of non-demonstrative inferencing (see Rolf 2013: 13). Now, given that explicatures are recovered by means of pragmatic inferencing, they should be cancellable.^5^ In order to verify this assumption, let us take a look at some examples of explicature cancellation, beginning with explicit cancellation:

(5) a. Some of the students passed the exam.
  b. Actually, all of them passed the exam.

When sentence (5a) is used to convey the explicature that some but not all of the students passed the exam, this explicature can be cancelled by the amendment of the cancellation clause in (5b) without giving rise to contradiction. The same holds for the following example:

(6) a. Tom and Jane are married.
  b. But they are not married to each other.

Assuming that sentence (6a) is used to convey the explicature that Tom and Jane are married to each other, this explicature may be cancelled by the amendment of the cancellation clause in (6b) without giving rise to contradiction. However, sometimes the explicit cancellation of explicatures seems to be rather difficult. To take one example:

(7) a. I haven’t eaten breakfast.
  b. #But I have eaten breakfast today.

Suppose that someone who is asked if she is hungry replies (7a), thereby explicating that she has not eaten breakfast that day. In this case, it is difficult to cancel the explicature on the grounds that it would be odd to amend the cancellation clause in (7b). But why is it difficult to cancel the explicature in question? In my opinion, this is due to the fact that (7a) semantically entails that the speaker has not eaten breakfast on the day of utterance. Accordingly, the attempt to cancel the explicature results in a contradiction.

So let us assume that there are explicatures that are semantically entailed. Given that such explicatures logically follow from the sentences they are based on, it is rather difficult to cancel them explicitly. Nevertheless, they can be cancelled contextually. In order to accomplish a contextual cancellation, it is necessary to find a situation in which the utterance would simply not carry the explicature. Suppose again someone uttering (7a), thereby explicating that she has not eaten breakfast that day. It is quite easy to imagine a situation where the utterance would not carry this explicature; for example, a situation in which someone is complaining about a weekend she spent in a sleazy hotel. In this case, an utterance of (7a) would carry the explicature that the speaker has not eaten breakfast there.

Summing up, we have seen that explicatures are cancellable in that they pass the Gricean cancellability test. Although it is sometimes rather difficult to cancel them by means of explicit cancellation, it is possible to cancel them by means of contextual cancellation. However, several objections to the cancellability of explicatures have been raised in recent years. The aim of the following section is to shed some light on these objections and to demonstrate that they are based on a misinterpretation of the Gricean cancellability test.

## Objections to cancellable explicatures

One thing should be understood from the start: the view that explicatures cannot be cancelled can be traced back to Capone (2003, 2009, 2013, 2015) and has been taken up particularly by Burton-Roberts (2005, 2010, 2013). First and foremost, this view is based on two general objections to the cancellability of explicatures. The first objection pertains to the logical structure of discourse. Given that explicatures rescue fragments of discourse from logical absurdity, it is claimed that their cancellation “would amount to returning to the problems which, in the first place, necessitated the explicature” (Capone 2013: 136). In other words, explicatures cannot be cancelled without impairing the logical structure of discourse. To take one example, imagine a situation in which someone is asked whether Karen is ready to leave for the airport:

(8) a. Karen is ready.
  b. #But Karen is not ready to leave for the airport.

By uttering (8a) the speaker conveys the explicature that Karen is ready to leave for the airport. However, the same speaker cannot cancel this explicature by means of (8b) without impairing the logical structure of discourse on the grounds that the cancellation of the explicature would amount to the discourse fragment which necessitated the explicature.^6^

The second objection to the cancellability of explicatures relies on the fact that explicatures are intended by the speaker and recognized as intended by the hearer. Since an intention implemented in an act of utterance cannot be unimplemented, there is no way out for the speaker who is committed to the proposition explicitly communicated by her utterance (see Burton-Roberts 2010: 138). Consider once again (8a) where the speaker conveys the explicature that Karen is ready to leave for the airport. Given that this explicature is intended by the speaker and recognized as intended by the hearer, it cannot be cancelled without giving rise to contradiction; intending to convey *X* and intending to convey *not*-*X* results in a contradiction. Either the speaker intends to convey *X* or she intends to convey *not*-*X*, but not both.^7^

As we have seen, Capone and Burton-Roberts maintain that we must take into account aspects such as the logical structure of discourse and the speaker’s communicative intentions in order to obtain the correct results when applying the cancellability test. However, I argue that placing the (explicit) cancellability test in actual discourse situations is a rather dubious step on the grounds that meanings are not cancelled by the speaker, but by the pragmatician who employs the cancellability test as a diagnostic tool. As said above, both parts of the cancellability test are nothing else but thought experiments; on the one hand, we have to imagine a cancellation clause which cancels the explicature; on the other hand, we have to imagine an utterance context in which the utterance does not give rise to the explicature. To that effect, the objections raised by Capone and Burton-Roberts turn out to be unwarranted. Neither the logical structure of discourse nor the speaker’s intentions matter for issues of cancellability.

Apart from that, Capone and Burton-Roberts both ignore that explicatures are contextually cancellable. Although there are cases in which an explicature is not explicitly cancellable, it is contextually cancellable for there are always other imaginable utterance contexts by which the explicature at hand is cancelled. As a matter of fact, this view on the cancellability of explicatures is in accordance with the suggestions made by Seymour on the cancellability of conversational implicatures:

The fact that a particular implicature cannot be canceled from a particular context of use is compatible with its cancelability within different contexts of use. Particularized conversational implicatures may be difficult to avoid in a particular context of utterance, but the very same act of saying involved in them could have been made in quite a different particularized context of utterance, and this is all we need to argue that conversational implicatures are cancelable. (Seymour 2010: 2871)

All in all, we have seen that the objections to the cancellability of explicatures seem to be unwarranted. Since issues of cancellability have nothing to do with actual discourse situations, these objections are based on a misinterpretation of the Gricean cancellability test.

## Conclusions

Within the Gricean framework only what is conversationally implicated is cancellable, whereas what is conventionally implicated and what is said cannot be cancelled without giving rise to contradiction. In this paper, we have seen that explicatures, which replace the notion of what is said in the relevance-theoretic framework, are cancellable on the grounds that they are recovered by means of pragmatic inferencing which is non-demonstrative by its very nature. Apart from that, we have seen that the objections to the cancellability of explicatures seem to be unwarranted. Once we acknowledge that issues of cancellability have nothing to do with actual discourse situations, the cancellability test turns out to be a coherent test and is easier to handle.

## Footnotes

1 For the first time, Grice makes use of the cancellability test in his paper *Meaning* where he gives two examples that demonstrate the explicit cancellability of non-natural meanings (see Grice 1957: 377–378). For the second time, the cancellability test is mentioned by Grice in *The Causal Theory of Perception* where he provides a short overview of his theory of conversation and relies on the cancellability test to distinguish between semantic presuppositions, conventional implicatures and conversational implicatures (see Grice 1961: 128). For the third time, the cancellability test is mentioned in *Logic and Coversation* where Grice employs this test to distinguish between conventional and conversational implicatures (see Grice 1975: 57).

2 Accordingly, explicit cancellations are not performed by speakers, but by pragmaticians who carry out the cancellability test.

3 See Akerman (2015), Blome-Tillmann (2013), Borge (2009), Dahlman (2013) and Haugh (2013).

4 As we will see, explicatures, which have to be distinguished from conversational implicatures, also pass the cancellability test. This is in line with Crimmins' critical remarks on the cancellability test. As he points out, cancellability “is not a property exclusively of pragmatic implicatures” (Crimmins 1992: 23).

5 As Carston points out: “the pragmatically inferred elements of an explicature can be explicitly cancelled without contradicting the encoded content of the utterance” (Carston 2010: 232).

6 According to Bach, sentence (8a) does not express a complete proposition, but merely a propositional radical. What distinguishes a complete proposition from a propositional radical is that the latter “lacks at least one constituent needed for it to be true or false” (Bach 2006: 437).

7 See also Feng (2013).
